# Reduced serum cholinesterase is an independent risk factor for all-cause mortality in the pediatric intensive care unit

**DOI:** 10.3389/fnut.2022.809449

**Published:** 2022-11-24

**Authors:** Chaoyan Yue, Chunyi Zhang, Chunmei Ying, Hua Jiang

**Affiliations:** Obstetrics and Gynecology Hospital of Fudan University, Shanghai, China

**Keywords:** cholinesterase, mortality, pediatric, ICU, risk factors

## Abstract

**Objective:**

Our aim was to assess the relationship between serum cholinesterase levels at intensive care unit admission and all-cause mortality in the pediatric intensive care unit.

**Methods:**

We used the pediatric intensive care unit database (a large pediatric intensive care database in China from 2010 to 2018) to conduct a retrospective analysis to evaluate the serum cholinesterase levels at intensive care unit admission of 11,751 critically ill children enrolled to the intensive care unit. We analyzed the association between serum cholinesterase and all-cause mortality. Adjusted smoothing spline plots, subgroup analysis and segmented multivariate logistic regression analysis were conducted to estimate the relative risk between proportional risk between serum cholinesterase and death.

**Results:**

Of the 11,751 children, 703 (5.98%) died in hospital. After adjusting for confounders, there was a negative association between serum cholinesterase and the risk of death in pediatric intensive care unit. For every 1,000 U/L increase in serum cholinesterase, the risk of death was reduced by 16% (adjusted OR = 0.84, 95% CI: 0.79, 0.89). The results of sensitivity analysis showed that in different stratified analyses (age, intensive care unit category, albumin, alanine aminotransferase, creatinine, neutrophils), the effect of serum cholinesterase on all-cause mortality remained stable.

**Conclusion:**

After adjusting for inflammation, nutrition, and liver function factors, cholinesterase reduction is still an independent risk factor for pediatric intensive care unit all-cause mortality.

## Introduction

Cholinesterases are divided into acetylcholinesterase, which is found in red blood cells and glial tissue, and “pseudocholinesterase,” also known as butylcholinesterase or serum cholinesterase, which is found in the serum and synthesized in the liver (1–4). Serum cholinesterase (ChE) is an alpha-glycoprotein that is released into the plasma immediately after synthesis and accounts for 90% of soluble cholinesterase in the blood (5–7). Serum cholinesterase is a surrogate marker of liver function and nutrition, reflecting the liver's ability to synthesize protein, and its activity is usually higher in men than in women and declines progressively with age. The half-life of cholinesterase in serum is 11 days, which is shorter than that of albumin (2, 8) and is therefore one of the sensitive indicators of nutritional status in clinical practice (9, 10).

Cholinesterase levels are high in patients with nephrotic syndrome, diabetes, hyperthyroidism, fatty liver, dyslipidemia and obesity and low in patients with acute and chronic liver injury, cirrhosis, malignancy, malnutrition, stress and inflammation, sepsis and organophosphorus toxicity (2, 11–16). In addition to this, decreased serum cholinesterase activity is associated with severity and mortality in critically ill patients (17–21).

Although serum cholinesterase is readily available in routine blood chemistry, previous studies have focused only on adults and the relationship regarding the prognosis of serum cholinesterase in pediatric intensive care unit patients has not been reported in the literature. In this study, we will investigate serum cholinesterase levels at admission in pediatric patients and assess the relationship between serum cholinesterase and all-cause mortality in pediatric intensive care unit patients.

## Methods

### Subjects

We performed a retrospective analysis using the Pediatric Specialty Intensive Care (pediatric intensive care unit) database, selecting 11,751 critically ill children with comprehensive laboratory test results. PIC (Pediatric Intensive Care) is a large pediatric-specific, single-center, bilingual database containing information related to children in the intensive care unit at the Children's Hospital of ZheJiang University School of Medicine, China, from 2010 to 2018. The pediatric intensive care unit database includes vital sign measurements, medications, laboratory measurements, fluid balance, diagnosis codes, length of stay, survival data, and more (22). The laboratory indicators we included are the results of the first inspection after admission to the intensive care unit. Inclusion criteria: Patients with complete records of demographic and clinical test results. This information includes gender; age; vasoactive drugs; first care unit; cholinesterase; lactate (LAC); neutrophils; glucose (GLU); cystatin C; blood urea nitrogen (BUN); creatinine (Cr); uric acid (UA); albumin (Alb); Total cholesterol (Tcho); triglyceride(TG); hemoglobin; alanine aminotransferase (ALT); aspartate aminotransferase (AST); γ-glutamyl transpeptadase (GGT); absolute lymphocyte value; prothrombin time (PT) and platelet (PLT) results. First care unit including neonatal intensive care unit (NICU), surgery intensive care unit (SICU), pediatric Intensive Care Unit (PICU), cardiac intensive care unit (CICU). The different categories of ICU are all from the same children's hospital and all patients are children, including critically ill neonates, infants and children. Vasoactive drugs including dopamine hydrochloride injection, dobutamine hydrochloride injection, adrenaline hydrochlaride injection, isoprenaline hydrochloride injection, phenylephrine hydrochloride injection and norepinephrine bitartrate injection.

PIC database is a public database; this project was approved by the Institutional Review Board/Ethical Committee of the Children's Hospital, Zhejiang University School of Medicine (2019_IRB_052). The requirement for individual patient consent was waived because the project did not impact clinical care, and all protected health information was deidentified (22). We formally applied for access through the procedures recorded on the PIC website and PhysioNet, and we have signed a data usage agreement. We handle data responsibly and adhere to the principle of cooperative research.

### Statistical analysis

Data is expressed as mean (SD) for continuous variables and percentage (%) for dichotomous variables. Subgroup analyses examined the relationship between serum cholinesterase and the risk of all-cause mortality according to age, intensive care unit category, inflammation, nutrition, liver function and other indicators. Interaction tests in the logistic regression model were used to compare odd ratios between the subgroups analyzed.

Logistic regression models were used to examine the effect of serum cholinesterase and other variables on the occurrence of all-cause mortality. Multivariate regression models included other variables, including sex, age in months, intensive care unit category, vasoactive drugs, LAC; neutrophils; GLU; cystatin C; BUN; Cr; UA; Alb; Tcho; TG; hemoglobin; ALT; AST; GGT; absolute lymphocyte value; PT, and PLT. The risk associated with all-cause mortality is reported per 1,000 U/L of continuous serum cholinesterase. Data was analyzed with the use of the statistical packages R (The R Foundation; http://www.r-project.org; version 3.4.3). All *P* values for statistics were 2-tailed, and *P* < 0.05 was regarded as statistically significant.

## Results

Our study included 11,751 children, including 6,718 boys and 5,033 girls. Median age at intensive care unit admission was 8.28 months (Q1–Q3: 1.12–39.73), median length of stay in intensive care unit was 2.82 days (Q1–Q3: 0.93–9.73), and 5.98% percent (703 patients) died. The median serum cholinesterase concentration is 6,648 U/L (Q1–Q3: 4,897–8,320).

Tables 1, 2 describes the baseline characteristics of the subjects, including demographic characteristics and some laboratory test results that may be related to the occurrence of mortality.

**Table 1:** Baseline characteristics of the study participants.

	**Cholinesterase**			
**Clinical cutoffs**	**<5,000 U/L**	**5,000 ≤ ChE < 8,000 U/L**	**≥8,000 U/L**	***p*-value**
No. of participants	3,120	5,161	3,470	
Age (months), Median (Q1-Q3)	0.69 (0.07–8.46)	8.38 (1.51–42.71)	21.29 (6.97–57.26)	< 0.001
Vasoactive drugs				<0.001
0	2,555 (81.89%)	3877 (75.12%)	2,469 (71.15%)	
1	565 (18.11%)	1,284 (24.88%)	1,001 (28.85%)	
Intensive care unit category, *N* (%)				<0.001
NICU	1,787 (57.28%)	1,156 (22.40%)	108 (3.11%)	
SICU	238 (7.63%)	1,104 (21.39%)	1,127 (32.48%)	
CICU	223 (7.15%)	1,115 (21.60%)	1,159 (33.40%)	
PICU	463 (14.84%)	929 (18.00%)	450 (12.97%)	
General ICU	409 (13.11%)	857 (16.61%)	626 (18.04%)	
Gender, *N* (%)				0.988
Male	1,787 (57.28%)	2,947 (57.10%)	1,984 (57.18%)	
Female	1,333 (42.72%)	2214 (42.90%)	1,486 (42.82%)	
Mortality, *N* (%)				<0.001
0	2,750 (88.14%)	4,894 (94.83%)	3,404 (98.10%)	
1	370 (11.86%)	267 (5.17%)	66 (1.90%)	

**Table 2:** Characteristics of clinical laboratory results of study participants.

	**Cholinesterase**			
**Clinical cutoffs**	**<5,000 U/L**	**5,000 ≤ ChE < 8,000 U/L**	**≥8,000 U/L**	***p*-value**
Cholinesterase (U/L)	3,840.98 ± 892.70	6,522.77 ± 868.92	9,742.13 ± 1,937.10	<0.001
LAC (mmol/L)	3.44 ± 3.46	2.48 ± 2.17	2.02 ± 1.63	<0.001
Neutrophils ( × 10^∧^9/L)	7.48 ± 6.52	5.96 ± 5.32	4.53 ± 4.17	<0.001
Alb (g/L)	31.56 ± 6.11	39.45 ± 5.41	44.14 ± 4.49	<0.001
Hemoglobin (g/L)	125.57 ± 40.53	123.31 ± 30.84	122.03 ± 18.12	<0.001
Tcho (mmol/L)	2.37 ± 1.18	3.37 ± 1.22	4.05 ± 1.09	<0.001
TG (mmol/L)	1.19 ± 1.43	1.24 ± 1.32	1.39 ± 1.00	<0.001
PT (s)	16.60 ± 9.03	13.26 ± 5.07	12.11 ± 4.92	<0.001
Lymphocyte ( × 10^∧^9/L)	3.44 ± 2.60	4.01 ± 2.65	4.59 ± 2.79	<0.001
PLT ( × 10^∧^9/L)	237.79 ± 149.21	311.92 ± 141.05	348.02 ± 124.79	<0.001
GLU (mmol/L)	5.86 ± 3.34	6.39 ± 3.34	6.39 ± 2.94	<0.001
Cystatin C (mg/L)	1.50 ± 0.68	1.18 ± 0.59	0.97 ± 0.38	<0.001
BUN (μmol/L)	5.17 ± 4.77	4.20 ± 3.66	4.05 ± 2.15	<0.001
UA (μmol/L)	318.29 ± 214.05	293.31 ± 160.52	275.77 ± 117.77	0.001
Cr (μmol/L)	64.64 ± 27.77	51.82 ± 21.24	45.99 ± 13.79	<0.001
ALT (U/L)	39.11 ± 73.05	33.40 ± 58.47	28.71 ± 46.18	<0.001
AST (U/L)	179.43 ± 786.72	112.21 ± 737.84	78.00 ± 681.86	<0.001
GGT (U/L)	120.39 ± 131.25	82.15 ± 148.48	32.51 ± 101.78	<0.001

LAC, lactate; GLU, glucose; BUN, blood urea nitrogen; Cr, creatinine; UA, uric acid; Alb, albumin; Tcho, Total cholesterol; TG, triglyceride; hemoglobin; ALT, alanine aminotransferase; AST, aspartate aminotransferase; GGT, γ-glutamyl transpeptadase; PT, prothrombin time; PLT, platelet.

The effect of age on serum cholinesterase was observed in Table 1, so we adjusted for age as a confounding factor when studying the effect of cholinesterase on all-cause mortality. Sensitivity analyses were performed to verify the effect of cholinesterase on all-cause mortality in patients of different ages. The results in Table 3 show that, after stratification by age, serum cholinesterase was a protective factor for all-cause mortality across age groups without interaction (*p*-value for interaction=0.241). Other subgroup analyses are shown in Table 3. We found that according to intensive care unit type, neutrophils, albumin, ALT, Cr, the effect of serum cholinesterase on all-cause mortality remained consistent between the subgroups analyzed. In the different subgroups, the incidence of all-cause death in the pediatric intensive care unit decreased significantly with increasing serum cholinesterase concentrations. It should be noted that the protective effect of serum cholinesterase on mortality is reduced in neonates, inflammatory conditions, and renal impairment, but the overall protective trend still exists.

**Table 3:** Sensitivity analysis between cholinesterase per 1,000 U/L and mortality.

**Sub-group**	**n**	**OR**	**95% CI**	***p*-value**	***p*-value for interaction**
Age (months)					0.241
>12	5,228	0.89	(0.81, 0.97)	0.0119	
(1, 12)	3,703	0.80	(0.72, 0.89)	<0.0001	
≤ 1	2,820	0.89	(0.75, 1.05)	0.1511	
Intensive care unit category					0.2637
NICU	3,051	0.96	(0.81, 1.14)	0.6165	
SICU	2,469	0.84	(0.69, 1.03)	0.0903	
CICU	2,497	0.72	(0.57, 0.92)	0.0080	
PICU	1,842	0.82	(0.73, 0.91)	0.0004	
General ICU	1,892	0.90	(0.81, 1.01)	0.0724	
Alb (g/L)					0.1189
5.1–36	3,912	0.87	(0.80, 0.96)	0.0035	
36.1–42.7	3,854	0.76	(0.69, 0.84)	<0.0001	
42.8–68.5	3,985	0.88	(0.76, 1.00)	0.0576	
ALT (U/L)					0.9329
≥40	1,978	0.87	(0.79, 0.97)	0.0090	
< 40	9,655	0.84	(0.78, 0.91)	<0.0001	
Cr (μmol/L)					0.0137
5–40.9	3,567	0.82	(0.73, 0.91)	0.0002	
41–55.9	4,055	0.74	(0.65, 0.84)	<0.0001	
56–140	3,889	0.93	(0.84, 1.03)	0.1432	
Neutrophils ( × 10^∧^9/L)					0.1174
0.01–2.87	3,854	0.80	(0.71, 0.92)	0.0010	
2.88–5.91	3,913	0.83	(0.73, 0.94)	0.0025	
5.92–63.08	3,882	0.91	(0.84, 0.99)	0.0332	

Table 4 shows the results of the multiple regression of serum cholinesterase on the risk of death. Without adjustment for confounders, the risk of death was reduced by 27% for each 1,000 U/L increase in serum cholinesterase (OR = 0.73, 95% CI: 0.70, 0.75); after full adjustment for confounders, the risk of death was reduced by 16% for each 1,000 U/L increase in serum cholinesterase (adjusted OR = 0.84, 95% CI: 0.79, 0.89). Serum cholinesterase was grouped according to cutoff values of 5,000 and 8,000, and after full adjustment for confounding, there was a statistically significant 38% reduction in the risk of death for serum cholinesterase at 5,000–8,000 U/L and an 72% reduction in the risk of death for serum cholinesterase at >8,000 U/L compared with those with serum cholinesterase < 5,000 U/L.

**Table 4:** Individual effect of serum cholinesterase on all-cause mortality.

**Exposure**	**Incidence, *n* (%)**	**Non-adjusted**		**Adjust model I**		**Adjust model II**	
		**OR (95% CI)**	***p* value**	**OR (95% CI)**	***p* value**	**OR (95% CI)**	***p* value**
Continuous cholinesterase per 1,000 U/L					
	703 (5.98%)	0.73 (0.70, 0.75)	<0.0001	0.75 (0.72, 0.78)	<0.0001	0.84 (0.79, 0.89)	<0.0001
Clinical cutoffs							
<5,000 U/L	370 (11.86%)	Reference		Reference		Reference	
(5,000, 8,000) U/L	267 (5.17%)	0.41 (0.34, 0.48)	<0.0001	0.42 (0.35, 0.50)	<0.0001	0.62 (0.49, 0.79)	0.0001
≥8,000 U/L	66 (1.90%)	0.14 (0.11, 0.19)	<0.0001	0.17 (0.13, 0.23)	<0.0001	0.28 (0.19, 0.41)	<0.0001

Adjust model I: Adjusted for gender, vasoactive drugs, age and intensive care unit category.

Adjust model II: Adjusted for gender, vasoactive drugs, age, intensive care unit category, cystatin C; UA; BUN; Cr; Alb; hemoglobin; GLU; ALT; AST; GGT; absolute neutrophil; absolute lymphocyte; PLT; LAC; TG; Tcho, PT.

## Discussion

Determining which predictors are associated with in-hospital mortality in the pediatric intensive care unit remains very challenging. Our study elucidated for the first time the relationship between serum cholinesterase levels and all-cause mortality in the pediatric intensive care unit, and after adjusting age, intensive care unit type, neutrophils, albumin, ALT, Cr, decreased serum cholinesterase was an independent risk factor for all-cause mortality risk in the pediatric intensive care unit.

It has been reported in the literature that low cholinesterase concentrations are associated with mortality in critically ill patients (14, 23). Low serum cholinesterase concentrations increase the risk of death in patients with other diseases such as acute myocardial infarction (19), acute heart failure (24), acute respiratory distress syndrome (25), stable coronary artery disease (26), and ischemic stroke (27). Reduced serum cholinesterase activity in patients with sepsis is associated with increased 30-day mortality in the EICU setting, even after adjusting for potential confounders, including acute multiorgan dysfunction. Each unit decrease in serum cholinesterase activity (KU/L) on EICU admission is associated with a 2.11-fold (95% confidence interval 1.37–3.27) increase in the risk of 30-day mortality (28). CHE concentration at admission may be a predictor of 30-day survival in elderly patients with acute ischemic stroke and patients with lower CHE concentrations have an increased risk of mortality during hospitalization (25).

Why are serum cholinesterase concentrations more sensitive than other candidate parameters? Serum cholinesterase levels are a pleiotropediatric intensive care unit biomarker reflecting a variety of factors including systemic inflammation, malnutrition, and hepatocellular injury, and therefore it is more closely associated with disease prognosis than are indicators of inflammation or nutrition alone.

The cholinergic anti-inflammatory pathway plays a key role in the systemic response to infection (29, 30). A decrease in blood BChE activity may be associated with severe systemic inflammation (31). The rapid (1–2 h) and intense decrease in BChE activity following an inflammatory challenge is recorded significantly earlier compared to routinely measured biomarkers of inflammation, and thus may serve as an early and effective marker of systemic inflammation (6). The sustained decrease in BChE activity observed in deceased patients suggests that serum cholinesterase is a new risk warning indicator in the intensive care setting, an indicator that allows simple and rapid screening of patients at risk for sepsis (6).

Cholinergic activation modulates the immune response and limits the pro-inflammatory process to a non-toxic range. Acetylcholine inhibits macrophage activation and pro-inflammatory cytokine release on the one hand, and promotes the synthesis and secretion of anti-inflammatory cytokines on the other, thereby attenuating the development of endotoxic shock (32). The abundant and rapid activity dynamics of serum BChE makes this enzyme a biochemical monitor of extrasynaptic cholinergic activity. The acetylcholine-serum cholinesterase homeostasis is disrupted during the acute phase of the immune response and imbalance in cholinergic homeostasis of the immune system is involved in individualized differences in patient prognosis (33). On the other hand, low cholinesterase levels are associated with an ineffective cellular immune response to bacteria, which may increase bacterial aggressiveness and more infectious complications in the low cholinesterase group regardless of the presence of remote infection at the surgical site (2, 8). Given that forebrain cholinergic neurons regulate innate immune responses and inflammation. A novel anti-inflammatory idea based on targeting forebrain cholinergic signaling in sepsis and other diseases is characterized by immune dysregulation, suggesting a promising clinical application of anti-acetylcholinesterase inhibitors in inflammatory conditions (34, 35).

Commonly used serum markers reflecting the nutritional status of patients include albumin, total cholesterol, cholinesterase, and total peripheral lymphocyte count (TLC). Nutritional status scoring systems based on these indicators include the controlled nutritional status (CONUT) score and the prognostic nutritional index (PNI). The CONUT score includes serum albumin and cholesterol concentrations as well as TLC, whereas the PNI includes serum albumin and TLC concentrations (36). Malnutrition may be a cause of respiratory muscle weakness and may increase the risk of aspiration due to dysphagia. Decreased serum albumin concentrations are associated with increased mortality in critically ill patients (6). Serum cholinesterase and albumin are both synthesized in the liver and have serum half-lives of 8–11 days and 12–17 days, respectively, so serum cholinesterase provides a more accurate assessment of pediatric intensive care unit nutritional status (37). Moreover, in patients receiving albumin supplements, the true synthetic function of the liver is masked, but easily assessed by monitoring serum cholinesterase. In cases where PT and serum albumin are not available, serum cholinesterase can play an important role as a marker of hepatic synthetic function (4).

Our results showed that after sensitivity analysis stratified by inflammatory indicators (neutrophils), nutritional indicators (albumin), kidney function indicators (creatinine) and liver function (ALT), serum cholinesterase had a consistent effect, and after adjusting for inflammation, nutrition, kidney function and liver function-related parameters, serum cholinesterase remained an independent risk factor for all-cause mortality risk.

At present, there is only one literature that reports the reference range of serum cholinesterase. The report points out that the reference ranges of children and adult women is 3,070–8,483 U/L, and the average is 5,687 U/I. The reference range for adult males is 4,687–9,116 U/L (38). In China, the reference range of adult serum cholinesterase is 5,000–12,000 U/L. For healthy newborns, liver function tests are not performed at the health care facility and therefore cannot be analyzed retrospectively from LIS data in the hospital. Similarly, in this study of the study population on patients in the pediatric intensive care unit, serum cholinesterase levels could not be used for the establishment of a reference range. Our data showed differences in cholinesterase levels across age in children in the pediatric intensive care unit, so in evaluating cholinesterase and all-cause mortality, we adjusted age for confounders, and after stratified analysis by age, cholinesterase had a stable and consistent effect in children of different ages.

This study also has some limitations. First, it was a single-center retrospective observational study. The effect of residual confounding factors cannot be completely excluded. The population studied is not the typical universal population of PICU patients, neoneates consists about 25%. Second, although we demonstrated that serum cholinesterase is a risk factor for mortality in pediatric intensive care unit patients, this study did not elucidate the clinical utility of cholinesterase as a therapeutic target during nutritional therapy and did not clarify whether altering serum cholinesterase concentrations by nutritional support reduces all-cause mortality in pediatric intensive care unit. Prospective clinical trials are needed to clarify whether correction of serum cholinesterase (ChE) concentrations by nutritional support such as oral or enteral immunonutrition can reduce all-cause mortality in pediatric intensive care unit. Third, serum cholinesterase concentrations are strongly influenced by dietary intake, but accurate dietary information for each patient before and after admission was not available in this study. Next, our study is based on a public pediatric intensive care database in which the children included are intensive care unit patients and therefore cholinesterase serum levels in healthy children are temporarily unavailable, however this does not affect the purpose of this paper and our findings apply only to children in pediatric intensive care units. In the subgroup analysis, Table 3 details the effect values for cholinesterase at different ages, in children with normal and abnormal liver function, in the presence of inflammation and in the absence of inflammation groups, and an interaction test was performed. Another limitation is that other risk factors that may affect mortality are not included, such as Vasoactive inotropic index (VIS) score, mechanical ventilator requirement, CRRT requirement. Finally, this study included only Chinese children and therefore requires careful interpretation before our data can be applied globally.

## Conclusion

In summary, decreased serum cholinesterase is an independent risk factor for all-cause mortality risk in the pediatric intensive care unit, serum cholinesterase can be used to identify high-risk patients at the time of pediatric intensive care unit admission, and serum cholinesterase can provide an accurate assessment of nutritional and hepatic synthetic capacity in pediatric intensive care unit patients, allowing us to implement early and appropriate nutritional interventions in daily practice, leading to a better prognosis.

## Data availability statement

Publicly available datasets were analyzed in this study. This data can be found here: http://pic.nbscn.org/.

## Ethics statement

The studies involving human participants were reviewed and approved by the Institutional Review Board/Ethical Committee of the Children's Hospital, Zhejiang University School of Medicine (2019_IRB_052). Written informed consent for participation was not provided by the participants' legal guardians/next of kin because: the requirement for individual patient consent was waived because the project did not impact clinical care, and all protected health information was deidentified.

## Author contributions

CYu analyzed the data, drafted the manuscript, and contributed to study design. CZ contributed to data collation. CYi and HJ made contribution to the conception, design, and revision of the revised manuscript. All authors read and approved the final manuscript.

## Funding

This work was supported by the program for National Natural Science Foundation of China (No. 81902131) and Shanghai Rising Stars of Medical Talents Youth Development Program [SHWRS(2020)_087]. Funding sources played no role in the design of the study and collection, analysis or interpretation of data, and played no role in the drafting, revision, or submission of the manuscript.

## Conflict of interest

The authors declare that the research was conducted in the absence of any commercial or financial relationships that could be construed as a potential conflict of interest.

## Publisher's note

All claims expressed in this article are solely those of the authors and do not necessarily represent those of their affiliated organizations, or those of the publisher, the editors and the reviewers. Any product that may be evaluated in this article, or claim that may be made by its manufacturer, is not guaranteed or endorsed by the publisher.
